# Characterization and Evolution of Mental Health Problems Attended to in a Telephone Helpline During the Lockdown and De-Escalation by COVID-19

**DOI:** 10.3389/ijph.2022.1604561

**Published:** 2022-06-20

**Authors:** Alba Pérez-González, Sonia Benítez-Borrego, Jordi Garcia-Sicard, Andrés Cuartero, Silvia Ruiz-Torras, Joan Guàrdia-Olmos

**Affiliations:** ^1^ Faculty of Psychology and Education Sciences, Open University of Catalonia, Barcelona, Spain; ^2^ Research Group of Quantitative Psychology, University of Barcelona, Barcelona, Spain; ^3^ Emergency Medical System of Catalonia, Hospitalet del Llobregat, Spain; ^4^ Department of Social Psychology and Quantitative Psychology, University of Barcelona, Barcelona, Spain; ^5^ Institute of Neurosciences, University of Barcelona, Barcelona, Spain; ^6^ Universitat de Barcelona Institute of Complex Systems (UBICS), University of Barcelona, Barcelona, Spain

**Keywords:** mental health, COVID-19, emergency medical services, psychological distress, psychosocial intervention, help-line

## Abstract

**Objectives:** To picture the psychological impact on the general population consulting the Emergency Medical System (EMS) of Catalonia for psychological assistance due to the COVID-19.

**Methods:** Calls received to the 061 emergency phone number between the months of March and June 2020 (period of lockdown and de-escalation) were analyzed. The reason, most prevalent psychological symptoms, presence of psychological antecedents, and type of intervention that was carried out were analyzed.

**Results:** A total of 2,516 calls were analyzed. Weeks 6, 7, 8 and 9 of lockdown saw the highest volume of calls (298, 314, 282 and 290 daily calls, respectively). The main profile of the affected person was women, under the age of 50 who are responsible for others. Psychologically, they present symptoms of depression (7.33%) and anxiety (39.44%). The greatest impacts on mental health throughout lockdown seem to be related to an increase of interpersonal conflict (8.8% < 11.2%), work-related problems (1.7% < 4.6%), and problems of psychological distress (6.5% < 17.0%).

**Conclusion:** The information obtained enables us to better understand the possible evolution of the impacts on mental health derived from the lockdown.

## Introduction

Lockdown during the COVID-19 pandemic has undoubtedly impacted people’s mental health. More specifically, high levels of worry, stress, and hopelessness have been observed [1–3], poorer quality of sleep [3–5] and symptoms of depression [3, 6, 7], among others. In some cases, it has produced or exacerbated certain psychological problems (depression, anxiety, and stress) [4].

Several studies suggest that this psychological impact has been different in men and women, with it being more negative in women [1, 5, 8]. More symptoms of depression have been observed in women [1, 5], as well as higher levels of irritability, mood swings, and twice the rate of anxiety attacks [5]. Evidence of differences in age has also been found. For example, some studies indicate that young people (19–30 years old) have been more affected by lockdown [1].

Regarding the risk or vulnerability factors, it is worth highlighting the processes of loss and grief. The families experienced grief without death rituals, in a solitary and unexpected way, perceived as unfair, insecure, and coexisting with other stressful factors [9]. At another level of risk and vulnerability, it is shown that excessive exposure to the media, living with chronically ill patients, and living with children under 12 years of age have been risk factors for the negative psychological impact of lockdown [1]. In addition, having had a previous mental disorder has been a risk of developing maladaptive, depressive, and anxious responses [6]. On the other hand, a low level of economic income has been associated with more depressive symptoms [6]. Regarding the stress response, family responsibilities increased the risk of developing stress symptoms, regardless of mental health status [6].

In relation to the reason for consultation to telephone psychological care services during lockdown, previous studies have shown as the most prevalent ones were the following: concerns about psychological symptoms (no COVID) (75.9%), fear or concern of infection, contagion and prognosis of the COVID-19 own or others (20.9%), as well as conflicts or complicated family situations (19.6%) [10].

Regarding the restrictions derived from lockdown, some important dates that should be highlighted in Catalonia are: strict home lockdown begins on 13 March 2020 until the end of April. Phase 0 of the de-escalation begins in May (children can go out accompanied, outdoor sports and takeaway restaurants). On May 11, some health regions of Catalonia go to phase 1 (opening of shops and restaurants with limited capacity, among others). Other health regions, went to phase 0.5, which had greater limitations (meetings in homes and the opening of terraces were still prohibited). On 25 May, regions in 0.5 advances to phase 1 and the rest advance to phase 2 where there is a greater opening of spaces and activities. As of June 15, the health regions are progressively moving towards phase 3 (flexibility of mobility and 50% capacity in public spaces).

The objective of this study was to describe the evolution in calls received to the 061/Salut Respon psychological assistance services of the Emergency Medical System of Catalonia (EMS), during lockdown and de-escalation. This description and picture constitute a sample of the possible evolution of the repercussions of lockdown at the mental health level of the population. For this, it is proposed to carry out a joinpoint trend analysis of the number of calls during the weeks of lockdown and de-escalation, as well as a cross-sectional descriptive analysis regarding the reason for consultation, psychological history, most prevalent symptoms, and psychological intervention collected in the analyzed calls.

Given the aforementioned antecedents, during the 2020 lockdown we expect to find similar results with regard to the psychological impact and mental health of people [1–10]. Consequently, specific objectives are proposed 1) to specify the most common profile in the affected people, and 2) to determine the evolution of the number of calls, specifying the type of intervention carried out, and the possible changes in the reason for consultation and in the most prevalent symptoms of the affected people. As a hypothesis, it is proposed that women will have had a more negative psychological impact, in the same way as young people. Likewise, at the level of symptoms, it will be expected a high frequency of depressive symptoms, anxiety and sleep problems, and the most serious symptoms (personality disorders, suicidal tendencies, psychotic disorders, substance use, etc.) will rise as the weeks of lockdown increase. Similarly, the symptoms of grief and traumatic experiences related to COVID-19 will continue throughout the weeks or will increase as the data on the prevalence of the virus evolve, and the relational conditions (conflicts at home, relationship problems, aggressions, etc.) as well as the social repercussions (labor, economic, neighborhood problems) will also rise as the period of lockdown increases, and they will decrease together with the isolation measures.

## Methods

### Sample

Retrospective analysis of the daily calls received to the 061 emergency phone number for psychological attention between the period of 25 March (date from which data are recorded) to 7 June 2021. During this time, an average of 34 calls per day were received. The period includes the lockdown phase (13 March to 1 May) and de-escalation (2 May to 7 June).

The psychological care was provided by a team of psychologists who were experts in emergency psychology and were already part of the EMS professional pool. As the psychological call center was implemented during the COVID-19 pandemic, all professionals in addition to having training and experience in emergency psychology, were trained in the psychological first aid model—6 Cs Model [11] adapted to their care for COVID-19.

### Procedure

During the period of lockdown and de-escalation, the calls received by the 061 EMS service regarding mental health issues were referred to the 061 PSCOVID psychological care service.

The hours of operation of the service was from 8 am to 11 pm, 7 days a week, with two psychologists per shift.

The calls were answered first by the EMS call manager, who forwarded the call according to its priority. All calls were recorded, and data confidentiality was always guaranteed.

The affected individuals were identified by telephone, collecting relevant data regarding clinical history, antecedents, reason for consultation, and the most prevalent symptoms. The categories of the reasons for consultation have been created based on a qualitative analysis of the reasons described by the professionals who answered the calls. The following categories emerged from this analysis: grief; COVID-19 (concerns about symptoms related to COVID-19); different COVID-19 symptom concerns (concerns related to psychological and/or physical symptoms other than COVID-19); interpersonal conflict (including from couple conflicts, discussions by both members normally without violence, to intra-family conflicts, both parents and children as well as other relatives, and gender violence; queries related to others (being able to be children, dependent persons, or queries related to third parties (other people); work problems; substance abuse; other reasons (social, neighborhood problems, suicide consultations); follow-up (in the case of second and third calls); combinations (various reasons); and no reason specified.

Minimal information was also collected on the type of intervention and assistance, differentiating between specific interventions (behavioral activation, breathing and relaxation techniques, thought restructuring, mediation/conflict management, accompaniment in grief, communication of bad news) and generic (active listening), normalization of reactions, psychoeducation, self-care guidelines, emotional ventilation).

If during the intervention it was necessary to activate other healthcare resources (sending an ambulance to the home, transfer to nursing/medicine, referral to Primary Care), this was collected as a binary response (activation/no activation).

Each psychology professional recorded this information in the EMS’s own registry program (SITREM), the healthcare tablet, and the individual registration form.

From these individual record sheets, which collected the information associated with an incident number, without identifying data and in a confidential way, the screening and analysis of the phone requests attended and of the interventions was carried out by psychologists trained for this purpose, based on a previously established category system for each variable.

### Data Analysis

First, a joinpoint trend analysis was performed using the number of calls answered as dependent variable and the weeks of lockdown and de-escalation as independent variable, showing the Week Percent Change (WPC) and their confidence intervals for each of the trends identified. Following the recommendations of the program based on the number of points observed, the maximum number of union points was set at 1. The model selection method used was the permutation test (setting the level of significance at 0.05, and the number of permutations at 4499).

Second, a descriptive analysis was conducted on the reason for the consultation, psychological history, most prevalent symptoms, and the intervention carried out.

Third, the descriptive analysis of the previous variables was expanded, focusing on their evolution throughout the weeks of lockdown, as well as the possible existence of differences between the lockdown and the de-escalation period. To do this, the *Chi-square* independence test is provided (noted that it is provided merely as descriptive data), as well as the Odds Ratio (OR) value and its 95% confidence interval (95% CI). In our case, the estimations of OR have only a descriptive purpose using de sampling estimation of the frequency of each category; offering de 95 IC as population estimation [12].

The statistical analysis of the data was carried out using the statistical package IBM SPSS Statistics version 27, R version 3.5.1 and Joinpoint Regression Program, version 4.9.0.1—February 2022; National Cancer Institute [13].

## Results

### Initial Descriptive Analysis

A total of 2,516 calls have been analyzed. The profile of the affected people corresponds to a median age of 47 years (IQR = 27), with a higher incidence between 36 and 55 years (39.80%), with 71.17% being women. In this sense, Table 1 shows the distribution of the reason for consultation of calls attended by 061 psychological consultants. The most prevalent reasons refer to concern for their own psychological symptoms not related to the COVID-19 disease, concern directly related to COVID-19, problems related to loss and grief, and consultations related to interpersonal conflicts.

**TABLE 1:** Description of the reason for consultation, psychological history, most prevalent symptoms, and psychological intervention carried out in the calls attended by 061 psychological consultancy (differentiating between periods of lockdown and de-escalation; Spain, 2020).

		Frequency	%
Reason for consultation	Different COVID-19 symptom concerns	312	13.23
	COVID-19	306	12.98
	Grief	280	11.87
	No reason specified	267	11.32
	Interpersonal conflict	255	10.81
	Follow-up	242	10.26
	Queries related to others	225	9.54
	Combinations: various reasons	188	7.97
	Other (social, neighborhood problems, suicide consultations)	140	5.94
	Work problems	84	3.56
	Substance abuse	59	2.50
Psychological history	No previous psychological history is specified	1719	74.61
	Previous psychological history is specified	585	25.39
Most prevalent symptoms	Symptoms of anxiety	990	39.44
	Other comorbidities	469	18.69
	No specific symptoms specified	359	14.30
	Mood symptoms	184	7.33
	Mixed symptoms of anxiety and depression	148	5.90
	Symptoms related to loss and grief	117	4.66
	Others	78	3.11
	Autolytic ideation	57	2.27
	Psychotic symptoms	36	1.43
	Personality problems	28	1.12
	Insomnia	25	1.00
	Traumatic symptoms	19	0.76
Psychological intervention carried out	No intervention specified	1274	55.51
	Concrete intervention	427	18.61
	Resource activation or referral	349	15.21
	Generic intervention	245	10.68
	Follow-up	697	27.70

In most cases, prior psychological antecedents were not specified.

Regarding the psychological symptoms (see Table 1), the affected people indicate anxiety symptoms, mood symptoms, and mixed anxiety-depression symptoms as the most prevalent.

On the other hand, with regard to the psychological intervention carried out, the type of intervention (specific to the symptoms and problems presented, or more generic interventions) is specified in those calls where the information was collected. Likewise, Table 1 also includes the cases in which the follow-up was carried out, as well as those for which an external resource was activated, either by referral of the case to primary care/mental health or through face-to-face assistance with resource mobilization (ambulance).

### Evolution of Calls During the Period Analyzed

**FIGURE 1:**
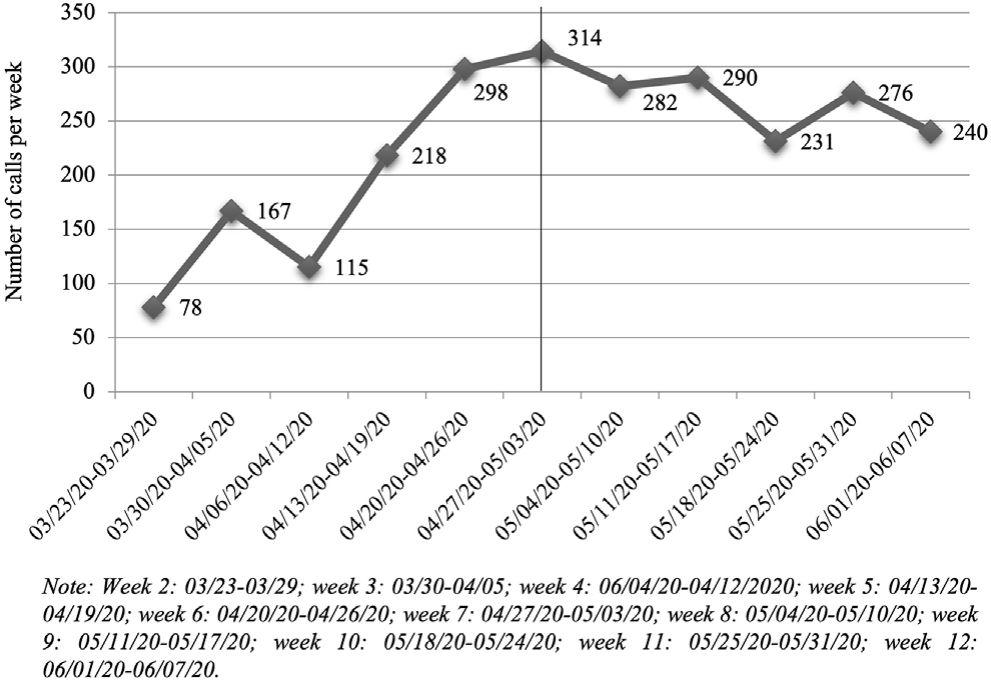
Scatter plot of the number of calls answered by 061 psychological consultants between 25 March and 7 June (differentiating between periods of lockdown and de-escalation; Spain, 2020).

As shown in Figure 1, (scatter plot of the number of calls from week 1 to week 12), the weeks of lockdown in which there were the highest number of calls were from the sixth to the ninth week. Likewise, the trend analysis identifies two periods (*p* < 0.05): from week 2–6 (where a progressive increase in calls throughout the weeks; WPC = 34.70), and from week 6–12 (detecting a less intense decrease in the number of calls; WPC = -2.65).

### Evolution of the Reason for Consultation Over the Weeks

**FIGURE 2:**
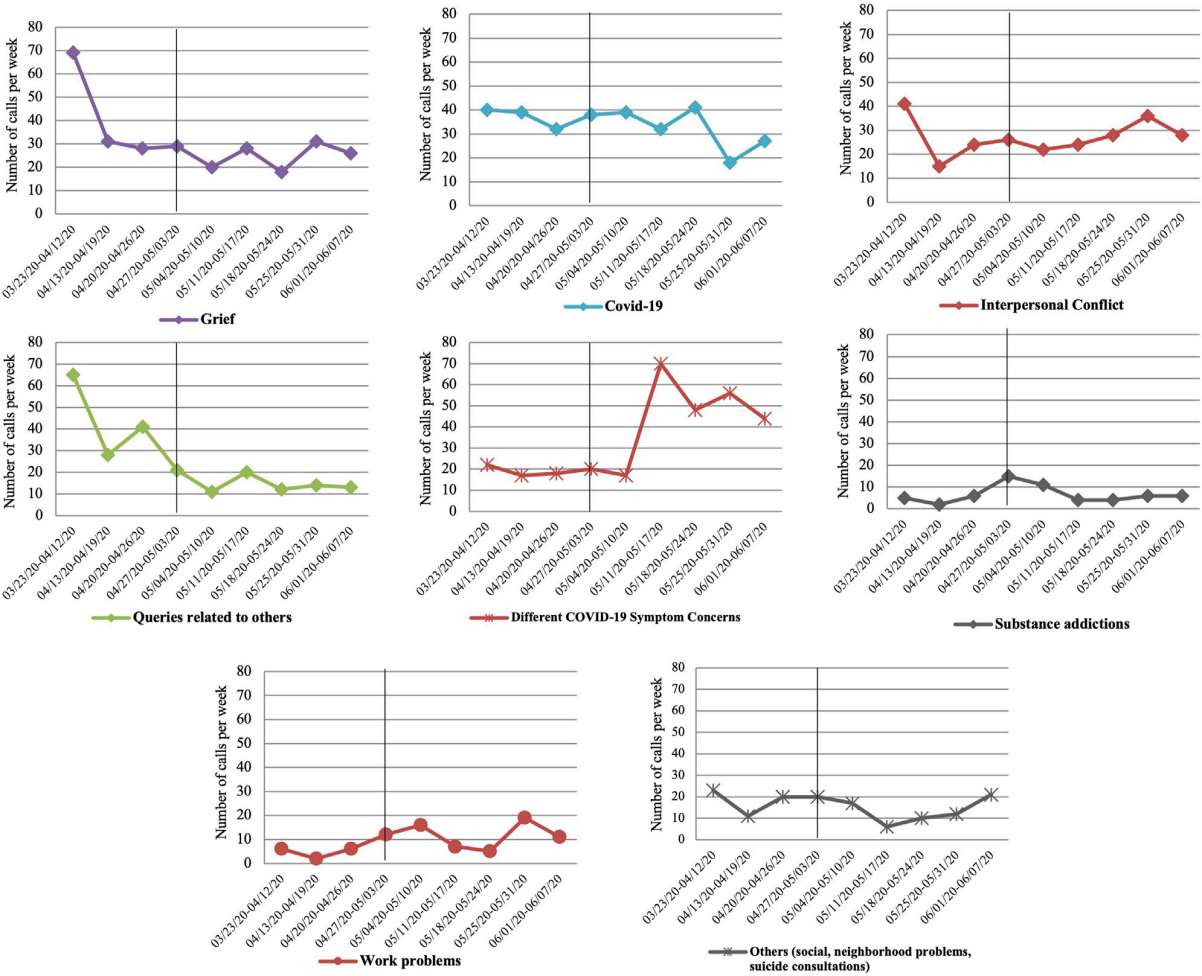
Evolution over the weeks of the reason for consultation mentioned in the calls answered (differentiating between periods of lockdown and de-escalation; Spain, 2020).

Regarding the relationship between the reason for consultation and its evolution over the weeks (χ^2^ = 524.74, df = 88, *p* < 0.001; V = 0.16), in the case of grief, a decrease in calls is observed after the first 3 weeks of lockdown, and relative stability is observed later (see Figure 2).

Regarding concerns related to COVID-19, the number of calls remains stable until week 10 and decreases in the eleventh and twelfth weeks.

In the case of consultations related to interpersonal conflict, calls related to concerns about their own psychological symptoms not related to COVID-19, as well as problems related to work, increase as the weeks of lockdown go on. The opposite effect is observed in the case of queries related to others (adults in charge, children, etc.). On the other hand, calls related to substance abuse problems (with a slight increase in the seventh and eighth weeks) and social and neighborhood problems, as well as suicide consultations, remain stable.

### Evolution of the Type of Intervention Over the Weeks

**FIGURE 3:**
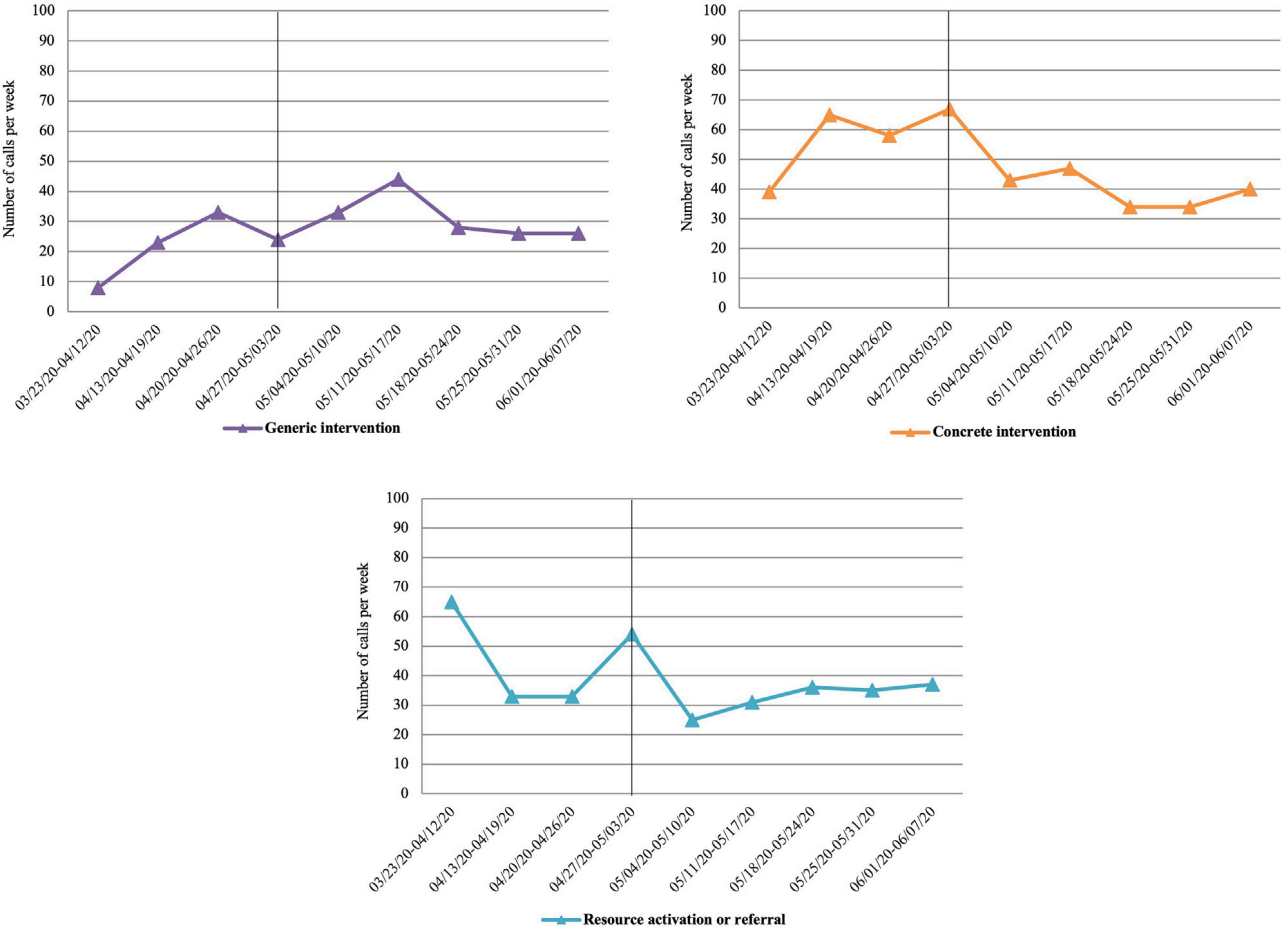
Evolution over the weeks of the type of intervention (differentiating between periods of lockdown and de-escalation; Spain, 2020).

Regarding the relationship between the type of intervention and its evolution over the weeks (χ^2^ = 113.51, df = 24, *p* < 0.001; V = 0.13), Figure 3 shows how as the weeks of lockdown increase, the more generic interventions increase slightly (up to the ninth week) and the specific interventions decrease slightly, with the number of activations of external resources remaining stable (referral, face-to-face intervention), with a slight rebound observed in the seventh week.

### Differences Between Lockdown and De-Escalation

Regarding the reason for consultation, differentiating between the lockdown and the de-escalation period (χ^2^ = 196.50, df = 11, *p* < 0.001; V = 0.28), on the one hand, in the de-escalation period there is a decrease in the number of calls related to grief problems and queries related to other dependents (children, the elderly, dependents, etc.). Meanwhile, calls related to concerns about their own symptoms other than COVID-19, as well as situations of interpersonal conflict and problems with work increase in the period of de-escalation with respect to lockdown (see Table 2).

**TABLE 2:** Description of the reason for consultation, most prevalent symptoms, and psychological intervention carried out in the calls answered (differentiating between periods of lockdown and de-escalation; Spain, 2020).

		Lockdown frequency (%)	De-escalation frequency (%)	OR (CI OR)
Reason for consultation	No reason specified	160 (14.5)	107 (7.6)	2.06 (1.58–2.70)*
	Grief	153 (13.9)	127 (9.0)	1.63 (1.26–2.11)*
	Queries related to others	152 (13.8)	73 (5.2)	2.92 (2.17–3.97)*
	COVID-19	138 (12.5)	168 (11.9)	1.06 (0.82–1.35)
	Follow-up	100 (9.1)	142 (10.1)	0.89 (0.67–1.17)
	Interpersonal conflict	97 (8.8)	158 (11.2)	0.76 (0.58–1.00)
	Different COVID-19 Symptom Concerns	72 (6.5)	240 (17.0)	0.34 (0.25–0.45)*
	Refuse assistance	71 (6.4)	81 (5.8)	1.13 (0.80–1.59)
	Others (social, neighborhood problems, suicide consultations)	69 (6.3)	71 (5.0)	1.26 (0.88–1.80)
	Substance addictions	24 (2.2)	35 (2.5)	0.87 (0.49–1.52)
	Work problems	19 (1.7)	65 (4.6)	0.36 (0.20–0.62)*
	Combinations: various reasons	47 (4.3)	141 (10.0)	0.40 (0.28–0.57)*
Most prevalent symptoms	Anxious symptomatology	384 (34.8)	606 (43.0)	0.71 (0.60–0.84)*
	Other comorbidities	188 (17.1)	281 (20.0)	0.82 (0.67–1.02)
	No specific symptoms specified	222 (20.1)	137 (9.7)	2.34 (1.85–2.97)*
	Mood symptoms	69 (6.3)	115 (8.2)	0.75 (0.54–1.03)
	Mixed anxious depressive symptoms	58 (5.3)	90 (6.4)	0.81 (0.57–1.16)
	Symptoms related to loss and grief	75 (6.8)	42 (3.0)	2.37 (1.59–3.58)*
	Others	41 (3.7)	37 (2.6)	1.43 (0.89–2.31)
	Autolytic ideation	23 (2.1)	34 (2.4)	0.86 (0.48–1.51)
	Psychotic symptoms	17 (1.5)	19 (1.3)	1.14 (0.56–2.34)
	Insomnia	13 (1.2)	12 (0.9)	1.39 (0.58–3.34)
	Personality problems	6 (0.5)	22 (1.6)	0.34 (0.11–0.88)*
	Traumatic symptoms	6 (0.5)	13 (0.9)	0.59 (0.18–1.66)
Psychological intervention carried out	Follow-up	577 (56.2)	687 (54.6)	1.07 (0.90–1.26)
	Concrete intervention	204 (19.9)	223 (17.7)	1.15 (0.93–1.43)
	Resource activation or referral	162 (15.8)	187 (14.9)	1.07 (0.85–1.35)
	Generic intervention	84 (8.2)	161 (12.8)	0.61 (0.45–0.81)*

Note: OR, odds ratio; CI OR, Confidence Interval at 95%; *OR statistically significant.

Thus, the analysis of the ORs reflects that the reasons most present in the lockdown period were grief and queries related to others, while, in the de-escalation period, concerns about their own psychological symptoms other than COVID-19 and work problems prevailed.

Regarding symptoms, differentiating between lockdown and the de-escalation period (χ^2^ = 94.37, df = 11, *p* < 0.001; V = 0.19), anxiety, mood, personality, anxious-depressive symptoms, and other comorbidities are observed to increase in the de-escalation period compared to the lockdown period. In contrast, loss-related symptoms and other non-specific symptoms decrease in the de-escalation period. On the other hand, psychotic symptoms, autolytic ideation, traumatic symptoms, and insomnia remain stable (see Table 2).

From the OR analysis, it is concluded that in the lockdown period, the symptoms related to grief are more present. On the other hand, in the de-escalation period, there seems to be a greater risk of anxiety symptoms related to personality patterns.

Regarding the type of intervention, differentiating between the lockdown and the de-escalation period (χ^2^ = 12.98, df = 3, *p* = 0.005; V = 0.08), generic interventions are observed to increase in the de-escalation period compared to the lockdown period (this type of intervention appearing as significant in the OR analysis), and the more specific interventions, as well as the activation of external resources, have a less significant increase (in terms of frequency) (see Table 2).

## Discussion

From the analyzes carried out, it was possible to establish that weeks 6, 7, 8 and 9 of lockdown saw the highest volume of calls. The main profile of the affected person was women, under the age of 50 who are responsible for others, and with symptoms of depression, anxiety, and sleep problems. The greatest impacts on mental health throughout lockdown seem to be related to an increase in situations of interpersonal conflict, work-related problems, and, above all, problems of psychological distress.

Firstly, the results obtained regarding the profile of the affected person are in line with previous studies [8], highlighting a female profile, under 50 years of age (observed characteristics of the sample), and with responsibilities for care of others (children, elderly people, etc.; being the “queries related to others,” one of the most prevalent query reasons). Some studies have found a younger age profile among people most psychologically affected by COVID-19 [1, 5]. In this study, the profile of the affected person seems to be associated with a higher age range of between 36 and 55 years and linked more to tasks related to care (“queries related to others” that includes children and elderly dependents). The psychological impact of this profile of affected person presents with a high frequency of symptoms of depression and anxiety and sleep problems, in line with other previous studies [5–7], which corresponds to an expected response to stress, so, highly nonspecific and not in the form of a particular psychopathology.

With regard to the second specific objective about the evolution in the number of calls to the emergency medical system related to mental health, the results indicate that there has indeed been a very significant increase compared to the same period of the previous year (an increase of over 58%). Specifically, between the months of April and May 2020, the increase in the volume of calls respect the same period of the previous year was from 110% to 150% [14]. These data are comparable as they cover the same months of the year and the same population, but with certain limitations and not directly generalizable in their content since the service that analyzed the demand at that time was not a specialized mental health service. Assessing it on a weekly basis, weeks 6, 7, 8 and 9 of lockdown were the weeks with the highest volume of calls, with a slight rebound in week 11. On the other hand, the results indicate a trend change from week 6. Looking at those dates, week 10, when calls decrease, coincides with the change to phase 0.5 or phase 1 of de-escalation, depending on the region. Week 6 in which the calls increase is not directly related to any significant event with regard to the evolution of the pandemic, but at a psychological level it does require a special significance when thinking in terms of the described phases of psychological response to disasters [15].

Regarding the variation in the reason for consultation, the results show an expected decrease in concerns related to the COVID-19 disease as the epidemiological data also improve, coinciding again from the tenth week. In the same way–and contrary to what had been hypothesized–, the decrease in consultations related to grief and loss, beyond the first weeks of lockdown, is relevant in the sense that as the pandemic advances and more people are affected, the informal systems of support and accompaniment in grief seem to perform their functions in most cases, requiring less and less specialized care.

In this sense, the greater repercussions in the area of mental health as the weeks of lockdown progress and the de-escalation occurs also seems to be related to an increase in situations of interpersonal conflict (conflicts at home, relationship problems, aggressions and victimizations, etc.), greater economic or work-related problems, and above all, or psychological distress of the population (as can be deduced from the high prevalence of reason for consultation related to concerns about their own psychological symptoms, not directly linked to COVID-19) [7, 16]. Thus, it is necessary to differentiate between the effects directly linked to COVID-19, its risk of infection or its negative effects, and another level of affectation based on the social repercussions that are derived. In both situations, the psychological response is expected.

As regards unforeseen results, it is worth noting the stability in the consultations related to the consumption and abuse of substances, with values even lower than in periods prior to the pandemic. This can be explained as a direct effect of lockdown, which reduces substance use in social settings and also disrupts the illicit drug market [17].

Regarding the evolution in the most prevalent symptoms of the affected people, the greatest impact on an emotional level seems to occur more in the de-escalation period than in lockdown [7]. As mentioned above, the psychological distress resulting from lockdown and, above all, its repercussions at the social level explain the increase in symptoms of a particularly anxious and/or depressive type, and at the personality level, specifically in those personality patterns that tend to be affected by interpersonal problems [16] (Cluster B). It is worth noting the increase in comorbid conditions as the weeks of lockdown increase.

Finally, regarding intervention, the results indicate that as the weeks progress, the type of interventions are more generic rather than more specific. This may be due to the comorbidity, both symptomatologic and the reason for consultation of the affected people, this being more diffuse and less specific due to the influence of other factors, especially of a social type (work, relational, economic) that seem to be present.

### Limitations

First, the cross-sectional approach used prevents establishing causal relationships between variables. Second, it is worth mentioning the size of the sample, which requires some caution in generalizing the results. However, the participants were not recruited for the study, rather all of them were treated by the emergency services without refining the sample, which makes them real cases. Another limitation of the sample, although it reflects social reality, is the high number of participating women. Regarding analysis of the information, the measurement of previous psychopathological history is not significant as it only collects whether or not this information has been specified during the call, but it could not be confirmed through, for example, the shared medical history. Likewise, the data on the personal characteristics of the subjects are very limited and this must be considered when drawing further conclusions in relation to the profile of the people treated. Finally, the information provided by each professional regarding the details of the intervention was minimal. We believe that the novelty of the situation (COVID-19), the lack of previous experience and, therefore, the lack of information on what the reasons for consultation and psychological repercussions could be, made the information provided focus more on these aspects than on the intervention itself. Further research is required to provide more information in this regard.

### Conclusion

This study provides significant data related to mental health and the profile of those affected during lockdown and de-escalation. The most frequent profile of those affected are women between the ages of 36 and 55 with anxiety and depression symptoms. A large part of the studies in recent months have focused on describing the clinical course of specific pathologies (eating disorders, traumatic spectrum disorders, suicidal ideation, behavior, etc.) and of different populations (children, adolescents, elderly); instead, we consider that community intervention and care plans and strategies should be defined focusing on a less specific but more frequent profile, such as the one highlighted in this study. Even more so considering that these are people with numerous responsibilities under their care (children, work, elderly) and that they may possibly find themselves neglected at times.

On the other hand, the results with regard to the most prevalent symptoms indicate that, at least during the period of lockdown and de-escalation, most of the affectation at the mental health level has been highly nonspecific (worries and sadness) and not in the form of concrete psychopathology. This is possibly more indicative of response and resistance to stress than of vulnerability to it. The differences found between the lockdown and de-escalation period support this hypothesis. It will be necessary to analyze the calls to the emergency services at later dates to determine the evolution at the mental health level.
